# Design and pilot results of a single blind randomized controlled trial of systematic demand-led home visits by nurses to frail elderly persons in primary care [ISRCTN05358495]

**DOI:** 10.1186/1471-2318-5-11

**Published:** 2005-09-08

**Authors:** Hein PJ van Hout, Giel Nijpels, Harm WJ van Marwijk, Aaltje PD Jansen, Petronella J van't Veer, Willemijn Tybout, Wim AB Stalman

**Affiliations:** 1 VU University medical center Amsterdam, Institute for Research in Extramural Medicine, Department of General Practice, Van der Boechorststraat 7, 1081 BT Amsterdam, The Netherlands

## Abstract

**Background:**

The objective of this article is to describe the design of an evaluation of the cost-effectiveness of systematic home visits by nurses to frail elderly primary care patients. Pilot objectives were: 1. To determine the feasibility of postal multidimensional frailty screening instruments; 2. to identify the need for home visits to elderly.

**Methods:**

*Main study*: The main study concerns a randomized controlled in primary care practices (PCP) with 18 months follow-up and blinded PCPs. Frail persons aged 75 years or older and living at home but neither terminally ill nor demented from 33 PCPs were eligible. Trained community nurses (1) visit patients at home and assess the care needs with the Resident Assessment Instrument-Home Care, a multidimensional computerized geriatric assessment instrument, enabling direct identification of problem areas; (2) determine the care priorities together with the patient; (3) design and execute interventions according to protocols; (4) and visit patients at least five times during a year in order to execute and monitor the care-plan. Controls receive usual care. Outcome measures are Quality of life, and Quality Adjusted Life Years; time to nursing home admission; mortality; hospital admissions; health care utilization.

*Pilot 1*: Three brief postal multidimensional screening measures to identify frail health among elderly persons were tested on percentage complete item response (selected after a literature search): 1) Vulnerable Elders Screen, 2) Strawbridge's frailty screen, and 3) COOP-WONCA charts.

*Pilot 2*: Three nurses visited elderly frail patients as identified by PCPs in a health center of 5400 patients and used an assessment protocol to identify psychosocial and medical problems. The needs and experiences of all participants were gathered by semi-structured interviews.

**Discussion:**

The design holds several unique elements such as early identification of frail persons combined with case-management by nurses.

From two pilots we learned that of three potential postal frailty measures, the COOP-WONCA charts were completed best by elderly and that preventive home visits by nurses were positively evaluated to have potential for quality of care improvement.

## Background

### Publishing the design of a study

Publishing the design and protocol of a study before results are available is important for several reasons. A published protocol allows easier comparison between what was originally intended and hypothesized and what was actually done, and it gives readers greater insight into the methodological quality of a study [1]. Furthermore, it has often been recognized that negative or adverse outcomes are less likely to be published [2]. Publishing the design of a study before its start announces the study will be undertaken, which encourages publication of the results and in any case informs researchers where they can find the data for inclusion in systematic reviews [1,1,2]. Thus, publishing a design article can prevent publication bias. In addition, publishing pilot results provides a better insight in the choices for particular instruments and interventions.

### Primary care and elderly

In the Netherlands all people are registered in a primary care practice and Primary Care Physicians (PCPs) act as gatekeepers to specialist care, whereas for example in the USA most persons are not registered in a primary care practice [3]. There are few barriers to primary care facilities in the Netherlands and in the Netherlands about 86% of older people contact their PCP yearly [4]. Older persons in primary care are, therefore, a good representation of the total older population at risk. Primary care is confronted with increasing numbers of frail elderly because of the aging of the population, and their wish to live independently for as long as possible. Frailty poses a complex problem for primary care. Up to about 20% of the elderly, defined here as those 75 years of age and over are vulnerable for further deterioration of functional abilities and quality of life accompanied by a substantial increased risk of institutionalization [5]. This implies an exploding need for care.

Primary care is insufficiently equipped for a potential explosion of care needs. GPs are often unaware of the health status and functional limitations of their elderly patients [6,7]. Several studies reported a considerable amount of undetected morbidity both among consulting and non-consulting patients [8,9]. Moreover, PCPs, as the medically responsible person, do not regard themselves suited for systematic management and long-term monitoring for chronic diseases and disabilities associated with frail health [10].

Proactive detection of care needs in elderly but still competent persons who do not explicitly seek help is at odds with the prevailing reactive paradigm in primary care. However, as perhaps many frail elderly are unaware of the types of help available, there is a need for care experiments with transmural collaboration among health professionals, which might increase the quality of (primary) care for frail persons at home.

### Earlier interventions

Systematic home visits to frail elderly by nurses can reduce mortality and nursing home admissions provided that a substantial number of home visits are paid and care plans are based on multidimensional assessments [11,12]. In addition, accumulating evidence shows that preventive home visits are mostly accompanied by a reduction of health care costs [13]. Both from patient (health gains) and societal (cost savings) perspective this is a desirable situation.

### Frailty and preventive mechanisms

Frailty is the result of reduced ability to maintain a physiological and psychosocial equilibrium, thereby increasing the risk of functional disability, temporary or permanent loss of the ability to cope, morbidity, and mortality [14-16]. Frailty is strongly associated with aging [17,18]. The potential preventive mechanisms of home visits comprise early detection of worsening health conditions and modifiable risk factors, enabling concerted actions with responsible health professionals to optimize treatment, improve life style and increase support for family caregivers to persevere informal care.

### Costs

Aging is costly. About one third of the health care expenditures in industrialized countries relate to persons 70 years or older [19]. Nursing homes, homes for the elderly and hospital beds are occupied mainly by elderly. Elderly are massive consumers of medication. Elderly consume most home care. When the number and portion of (frail) elderly increases the health care costs will explode. Among community dwelling elderly with usual care the median annual nursing home and hospital admission rate is 2.4% (range 0–40%) respectively 26% (range 5–56%). Among frail elderly median annual admission rates are 15% and 45% respectively [11,12].

### Objectives

To describe the design of an evaluation of the cost-effectiveness of systematic home visits by nurses to frail elderly primary care patients. Pilot objectives were to determine the feasibility of postal multidimensional frailty screening instruments and to identify the need for home visits to frail elderly. This article describes the background, design and pilot results.

## Methods

### Pilot 1: Selecting frail patients by postal questionnaires (Table 2)

**Table 2:** First Pilot: Selecting frail patients

Background:	Measuring frailty is subject to debate and various operational definitions were proposed [15]. For our purpose we sought a valid easy administrable self-report instrument.
Objective:	To determine the feasibility of multidimensional frailty screening instruments that could be sent by mail.
Methods:	After a literature search three multidimensional screening instruments were selected and tested in one general practice among all 75+ patients: 1) VES-13, 2) Strawbridge's frailty screen, and 3) COOP-WONCA charts. Feasibility was expressed in percentage complete item response [20–22]. Our goal was to identify the worst quarter. This point of departure was based on studies by Fried and Rockwood who reported between 20–30% of 75+ people to be frail according to their measures [14,17].
Results:	Of 116 patients 85 (81%) agreed to participate and 69 actually returned the questionnaire. The complete item response on the COOP-WONCA, Strawbridge screen, and VES-13 were 87%, 60% and 56% respectively. In order to identify a quarter of persons with the worst health on the COOP-WONCA, all persons were selected who scored in the worst quartile of at least two of the six charts (overall health ≥4; physical fitness ≥5; changes in health ≥4; daily activities ≥4; Feelings ≥3; social activities ≥3). This resulted in 23 persons who were further assessed at home by the RAI-HC. 90% had at least one chronic disease, two thirds had at least one ADL limitation, 60% had depressive symptoms (CESD>16) and 30% had cognitive impairment (MMSE<24) [37].
Conclusion:	The COOP-WONCA was the most feasible screener. Our selection rule identified a frail group. The geriatric assessment identified new potentially treatable problems.

COOP-WONCA = COOP functional health assessment charts – World Organization of Family Doctors

VES-13 = Vulnerable Elders Survey-13

RAI-HC = Resident Assessment Instrument – Home Care version

MMSE = Mini Mental Screen Examination

After a literature search three multidimensional screening instruments were selected and tested in one primary care practice among all 118 75+ patients: 1) VES-13, 2) Strawbridge's frailty screen, and 3) COOP-WONCA charts [20-22]. Feasibility was expressed in percentage complete item response. Our goal was to identify the worst quarter.

### Pilot 2: Exploring the potential for quality of care improvement of preventive home visits among elderly persons (Table 3)

**Table 3:** Second Pilot : Exploring the potential for quality of care improvement of preventive home visits among elderly persons.

Objective:	To identify the need and possible benefit of home visits for frail patients, PCPs and nurses.
Method:	The setting was a health center of 5400 patients with 3 PCPs and a practice nurse. Possible frailty was determined by the PCPs among their 75+ patients in the following cases: beginning dementia, active carcinoma, two or more medications for organ indication, treatments by two or more medical specialists, being 85+ and not contacted the PCP over the last three years, uncertainty regarding the ability to manage oneself, and all other persons the PCP felt it necessary to pay attention to. The nurses visited the patients and used an elaborate geriatric assessment protocol to identify psychosocial and medical problems. The nurses and the PCPs designed a care plan. The experiences of all participants were gathered by semi-structured interviews.
Results:	The participants (PCPs, nurses, patients) evaluated this approach positively. The PCPs gained better insight in medical and care situation of their elderly patients and experienced less work pressure. The nurses experienced better quality of care. The patients felt safer and more independent. The PCP also selected a number of healthy persons.
Conclusion:	Home visits by nurses were regarded by all to have potential for quality of care improvement. Point of concern was the inadequate selection of frail patients by the PCPs. Also, the assessment protocol used by the nurses provided no triggers on when actions should follow.

PCP = Primary Care Physician

The setting was a health center of 5400 patients with 3 PCPs and a practice nurse. Possible frailty was determined by the PCPs among their 75+ patients in the following cases: beginning dementia, active carcinoma, two or more medications for organ indication, treatments by two or more medical specialists, being 85+ and not contacted the PCP over the last three years, uncertainty regarding the ability to manage oneself, and all other persons the PCP felt it necessary to pay attention to. The nurses visited the patients and used an elaborate geriatric assessment protocol to identify psychosocial and medical problems. The nurses and the PCPs designed a care plan. The experiences of all participants were gathered by semi-structured interviews.

### Main study

#### Design

A randomized controlled trial in 33 primary care practices (55 primary care physicians) among frail 75+ patients at home who responded to a Health Screener, with 18 months follow-up. Frail persons living at the same address were randomized as one unit. PCPs are held blind for the group assignment. Block-randomization ensured equal numbers of intervention and usual care patients per practice. Random number tables were used by and independent person for randomization. The ethical committee of the VU medical center approved the study.

#### Study population main study

The PCPs provided the names and addresses of all their listed patients of 75 years or older and living at home. All persons received a health survey including the COOP-WONCA charts in order to identify the 20–25% elderly with the frailest functional health. The cut-offs per chart were based on a combination of reference data and our pilot data [22]. Inclusion and exclusion criteria are summarized in Table 1.

**Table 1:** Inclusion and exclusion criteria

Inclusion:	• Age 75 years and over and listed as general practice patient
	• Living at home
	• Frail: Self reported Health score in the worst quartile of at least two of six COOP-WONCA charts (scoring range: 1, excellent, to 5 very bad): Overall health ≥4; Physical fitness ≥5; Changes in health ≥4; Daily activities ≥4; Mental health ≥3; Social activities ≥3
Exclusion:	• Terminally ill as determined by PCPs
	• Persons with dementia symptoms according to MMSE or 7-minute screen
	• Living in residential homes.
	• Participating in other research projects

#### Intervention(s)

The scores of all persons who filled out the health survey and positively responded to the care offer were analyzed. The intervention consisted of 7 elements; (1) All frail persons and randomized to the intervention were contacted by one of eight trained nurses. In the first visit the nurses assess the health status and care needs by the Resident Assessment Inventory Home Care version (RAI-HC), a structured and computerized multidimensional geriatric instrument that enables direct and validated identification of problem areas [23,24]. The RAI-HC holds about 120 items and 30 domains of health and service needs (Table 5). It takes between 45 to 60 minutes to complete; (2) In our intervention the list of problems is discussed with the patient to determine whether additional care is needed.

**Table 5:** Case example of assessment by a nurse with the RAI-HC: triggered health risks

*Client Assessed Problem*	*Observed*	*Action undertaken earlier?*	*Relevant action now?*	*Immediate action?*	*Action later?*
1. ADL / Revalidation potential	X		X		
2. IADL / more formal care					
3. Health promotion					
4. Risk intramural admission					
5. Communication impairment					
6. Visual impairment					
7. Alcohol abuse					
8. Cognition					
9. Behavior					
10. Depression and Anxiety					
11. Abuse					
12. Social functioning					
13. Heart and lungs	X		X		
14. Dehydration	X			X	
15. Falls					
16. Nutrition					
17. Dental health					
18. Pain					
19. Bedsores	X	X			
20. Skin and food problems	X		X		
21. Compliance					
22. Vulnerable support system					X
23. Medication management					
24. Palliative care					
25. Preventive health	X				X
26. Psychofarmaca use	X	X			
27. Reduced service package					
28. Environment					
29. Feces incontinence					
30. Urinal incontinence catheter	X				

Therefore the nurse and the patient make a hierarchy of the problems; (3) The nurses design and execute individual suited care-plans that comply with patient priorities; (4) The nurses are case-managers and offer to visit the patients at least 5 times in a year in order to execute and monitor the care-plan, to evaluate whether the care-needs have changed and adapt the care/plan when needed; (5) The nurses also meet the PCPs on a regular basis to discus the care plans and to assure that medical actions are carried out by the PCPs; (6) To assure the quality of care, the nurses receive regularly educational updates and organize monthly meetings to discuss problematic cases. Two staff members supervise them. A national Dutch guideline on home care nursing of frail elderly patients was available [25]. This guideline was used to protocolize nurse interventions whenever possible; (7) The care plan is left at the patients' house to enable other visiting health professionals to take notice of and report on the care plan.

#### Outcomes and measurements

Table 4 provides an overview of all outcomes and measurements in the study.

**Table 4:** Measurement scheme

*Health screener*	*Instrument*	*T-1*	*T0*	*T1*	*T2*
				*6 months*	*18 months*
Functional Health status	COOP-WONCA	X		X	X
ADL & IADL	GARS	X		X	X
Cognitive decline	IQCODE self report	X			
Depressive symptoms	CES-D	X		X	X
Chronic diseases	Chronic diseases list	X			X
Mobility and Falls	Questionnaire	X		X	X
Body Mass Index	Questionnaire	X			
Weight change	Questionnaire	X			
Demographics	Questionnaire	X			
Behavioral problems	Questionnaire	X			
Incontinence	Questionnaire	X		X	X

*Main Outcomes*					
a. Health related Quality of life	SF36 + EQ5D		X	X	X
b. Hospital admissions	Patient + hospital database		X	X	X
c. (Days until) Institutionalization	PCP + nursing homes				X
d. (Days until) Mortality	Relatives + PCP				X
e. Health resource utilization	Self report + PCP + hospital + pharmacy databases		X	X	X

PCP = Primary Care Physician

GARS = Groningen Activity Restriction Scale

CES-D = Center for Epidemiological Studies – Depression Scale

IQCODE = Informant Questionnaire on Cognitive Decline in the Elderly

SF36 = Short Form 36 item version

EQ5D = EuroQuality of life

X = measurement

T-1 = pre randomization health screening

T0 = Measurement immediately after randomization

T1, T2 = Follow-up measurements

Outcomes are:

1. Health related quality of life as measured with the Short Form 36 (SF-36), and Quality Adjusted Life Years by health utilities based on Euroqol (EQ-5D) [26,27];

3. (Days until) institutionalization: Hospital stay, placement in nursing home or home for the elderly are surveyed and crosschecked at institutes;

4. (Days until) mortality as checked with the PCPs;

5. Direct costs as measured by patient questionnaires with three-monthly recall periods. These self-report data are supplemented by data from the centralized regional pharmacy database (medication use), regional hospital check, and nursing home checks. In case patients are not able to fill out the forms themselves a close relative will be approached (Table 4).

#### Sample size calculation

For an anticipated Health related Quality of Life benefit on at least two SF-36 domains with minimal relevant effect size Cohen's D = 0.5, 64 persons per group are required with a two sided alpha of 0.05 and 80% probability. Anticipating on an annual attrition of 20% (mortality, inability to respond, unwilling) 75 persons per group will be needed.

In a trial of 650 persons a reduction of 10% in hospital admission, institutionalization and mortality can be detected with a two-sided alpha of 0.05 and 80% probability (320 persons needed per group). Effect estimates are based on previous meta-analyses [11,12].

#### Data-analysis

According to the 'intention-to-treat' principle differences between intervention and usual care patients on mortality, hospitalization and nursing home placement (dichotomous outcomes) are tested by both chi-square tests and logistic regression analysis. Differences in time until these events will be analyzed with Cox-proportional hazard modeling. For quality of life (continuous outcome: SF-36, EQ-5D) General Linear Models (GLM), a technique for repeated measures is used to analyze group differences. Possible baseline differences in the outcome measures will be accounted for in GLM. Additional subgroup-analyses will be performed on types of recommendations in the care-plans.

Potential confounding and effect-modification is checked for sociodemographic characteristics, number and type of chronic disease, (I)ADL functioning (GARS), cognitive decline (IQCODE), mood (CES-D), behavioral problems (incontinence, sleep, agitation en aggression), medication use (centralized pharmacy data base) (Table 4) [28-30].

Quality of the data was assured by independent double checks of all forms. Also our institute employs a quality assurance policy. In this respect guidelines on all aspects of research were issued and all projects are subject to audits.

#### Economic evaluation

The economic evaluation will be performed alongside the randomized trial from a societal perspective. Data on resource use are collected in several ways: self report questionnaires, hospital and nursing home registration, and community pharmacy records. Only direct healthcare costs will be considered such as costs of consultations of the general practitioner, nursing home physician, medical specialist, hospitalizations, and medical department of the nursing home, and use of medication and medical aids. Medication data are retrieved from the centralized pharmacy files in the research region. If available, Dutch guideline prices are used to value resource use [31,32]. Otherwise, tariffs are used. Medication costs are valued using prices of the Royal Dutch Society for Pharmacy [33]. Contacts with GPs and referrals will be checked as well in GPs' patient information files.

##### Cost analysis

To compare costs between the two groups, confidence intervals for the differences in mean costs are calculated using bias-corrected and accelerated bootstrapping with 5000 replications. [34] For the cost-effectiveness analysis the difference in total costs between the intervention and usual care group are compared with the difference over 18 months in improvement of quality of life, reduced institutionalization, hospitalization and mortality. In addition, a cost-utility analysis will be done to assess the incremental costs per Quality Adjusted Life Years (QALY). Uncertainty around the cost-effectiveness and cost-utility ratios is calculated using the bias-corrected percentile method (5000 replications) and presented in a cost-effectiveness plane [35].

##### Patient outcome analysis

QALYs are calculated by multiplying the utility based on EQ-5D scores with the amount of time a patient spent in this particular health state [36]. Transitions between health states are linearly interpolated.

#### Recruitment

**Figure 1:**
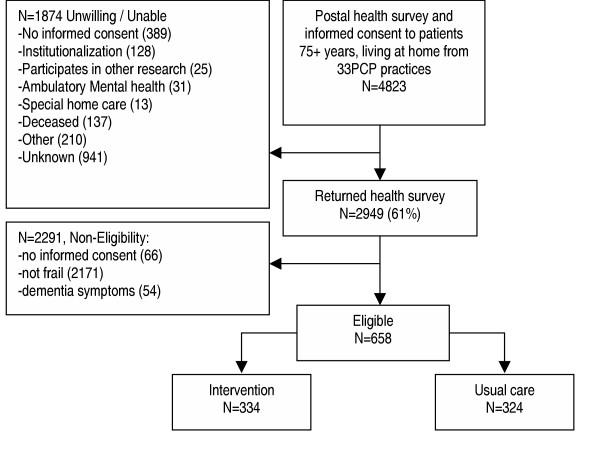
Flow chart PIKO.

The recruitment phase yielded a total of 33 PCP practices that were willing to participate. Inclusion started in spring 2003 and lasted until summer 2004. Figure 1 provides an overview of the recruitment and randomization. The health questionnaire was mailed to 4823 patients. Of the 2949 (61%) responders, 658 frail patients were detected and randomized.

##### Non-response

Females more often non-responded (41.3% versus 35.8%, X^2 ^= 13.4 p < 0.001), as did slightly older persons (83.5 versus 81.7 t = 13.8 p < 0.001).

##### Intervention

Eight nurses were trained in the use of the RAI-HC on laptops. The nurses were relatively unskilled in computer use so specific training was provided. Table 5 shows an example of a patient's problems list. Based on the detected problems the nurses design care plans.

## Discussion

In this paper we describe the design of a randomized cost-effectiveness trial of effect of preventive home visits by nurses to frail elderly primary care patients as well as the results of two pilot studies to determine the need and the feasibility instruments to select the frailest portion among elderly. The main study main study holds unique elements. The intervention concerns pro-active care that should guarantee timely detection of patients with frailer health, followed by structured nurse-led care focusing on patients. It focuses on the client as well as on the system around the client. The nurses use the Resident Assessment Instrument; a comprehensive geriatric assessment protocol that, by computerization, triggers health risks and, hereby, guides care planning.

The main study achieved a substantial response on the postal COOP-WONCA screening. In our pilot study, home visits by nurses were regarded to have potential for quality of care improvement. Point of concern was the inadequate selection of frail patients by the PCPs. Also, the assessment protocol used by the nurses provided no triggers on when actions should follow. In a second pilot study, the COOP-WONCA emerged as the most feasible postal screener compared to vulnerable elders survey (VES-13) and the Frailty screening list of Strawbridge. Our selection rule identified a frail group.

Limitations to the generalizability of our future findings are firstly the non-response to the mailed health survey. A number of persons (n = 100) responded but did not want to participate because of their good health. We remain uncertain about other non-responders being frailer and perhaps in greater need of nurse support. In future projects alternative means for health screening may be tested in older PCP patients such as contacting consulting patients. Secondly, our RAI assessment demanded a structured approach of computer skilled persons. The computer skills of the nurses were rather limited which led to extensive additional training. Some nurses had difficulties with the computerized assessments and disliked the structured format in which the assessment took place. Thirdly, we remain uncertain about the best frailty measure to identify persons that can benefit most from preventive actions. We selected the COOP-WONCA because of its broad health definition and very good feasibility. Last, whether PCPs remain blind for group assignment during he follow up remains to be seen. It is possible that the regular contacts with the nurses will reveal some of the assignments.

## Competing interests

The author(s) declare that they have no competing interests.

## Authors' contributions

HPJvH, GN and HWJvM designed the study. HPJvH drafted the article and all authors contributed to the final concept.

## Pre-publication history

The pre-publication history for this paper can be accessed here:


